# How to treat an articular base of distal phalanx fracture by transosseous indirect reduction

**DOI:** 10.1111/ans.70039

**Published:** 2025-02-11

**Authors:** Laurent Willemot, Timothy Marshall, Him Shun Yuen

**Affiliations:** ^1^ Department of Orthopaedic Surgery Launceston General Hospital Launceston Tasmania Australia; ^2^ College of Health & Medicine University of Tasmania Hobart Tasmania Australia

## Abstract

This case involves a patient with an articular base fracture of the distal phalanx, as indicated by the white arrows in the plain radiographs of the left hand: (a) posteroanterior, (b) oblique, and (c) lateral views. The fracture was treated using transosseous indirect reduction, a minimally invasive technique that provides stable fixation, facilitates early post‐operative recovery, and reduces surgical complications compared to conventional methods.

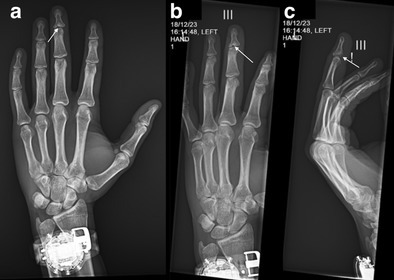

## Introduction

The distal phalanx of the middle finger is most commonly involved in fractures of the hand.^1^, ^2^ However, injuries involving comminuted intra‐articular fractures at the base of the distal phalanx are rare and present significant challenges for hand surgeons, given the difficulties associated with achieving accurate anatomical joint reduction and intra‐articular bone fragment stabilization.^3^, ^4^

Management options include both non‐surgical and surgical approaches. Non‐surgical management typically involves anatomic reduction followed by splinting and early hand therapy, with the goal of minimizing residual joint stiffness and promoting fracture healing.^2^ However, due to the intra‐articular extension of specific fractures, non‐surgical treatment often leads to suboptimal outcomes.^2^ Meanwhile, surgically, unstable distal phalanx fractures are conventionally managed through pinning of either the distal phalanx or the distal interphalangeal (DIP) joint using Kirschner wires (K‐wires).^5^ Despite its common use, this technique carries risks, including K‐wire migration, incomplete or lost reduction, pin tract infections, and arthropathies.^5^

To mitigate these complications, we propose an alternative method that combines indirect reduction—minimizing the risk of devascularization of articular fragments—and percutaneous screw fixation to enhance stability and reduce the risk of infection.^6^

This less invasive surgical technique involves indirect reduction using the blunt end of a reversed K‐wire as a bone tamp, followed by percutaneous stabilization with orthogonal screws. This approach not only provides stable fixation but also facilitates early joint mobility and recovery.

## Operative technique

**Fig. 1:**
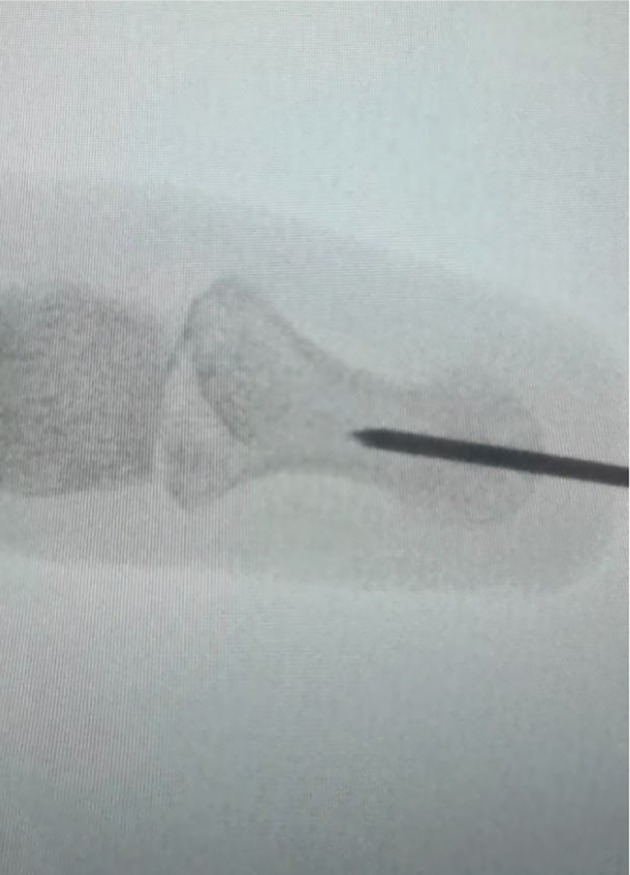
Intraoperative radiograph showing retrograde drilling of a 1.1 mm K‐wire through the tuft towards the joint surface.

**Fig. 2:**
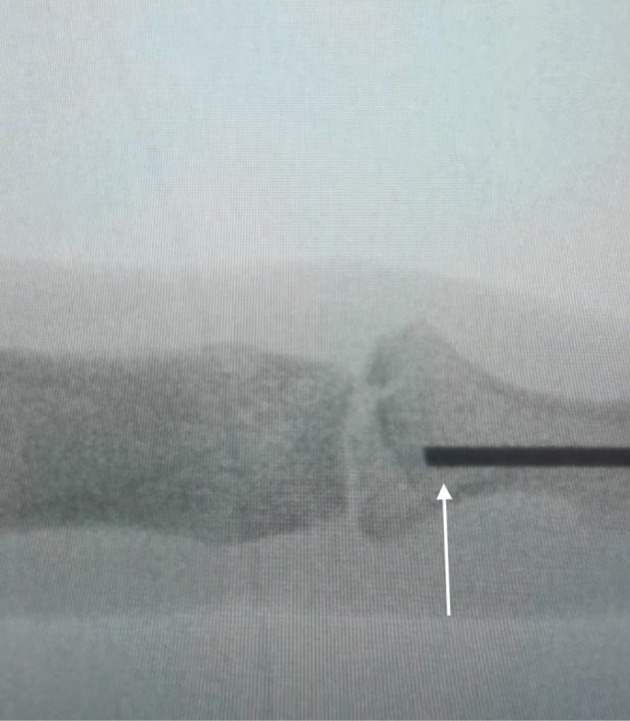
Intraoperative radiograph showing insertion of blunt end (white arrow) of K‐wire into the distal phalanx and applying it like a bone tamp.

**Fig. 3:**
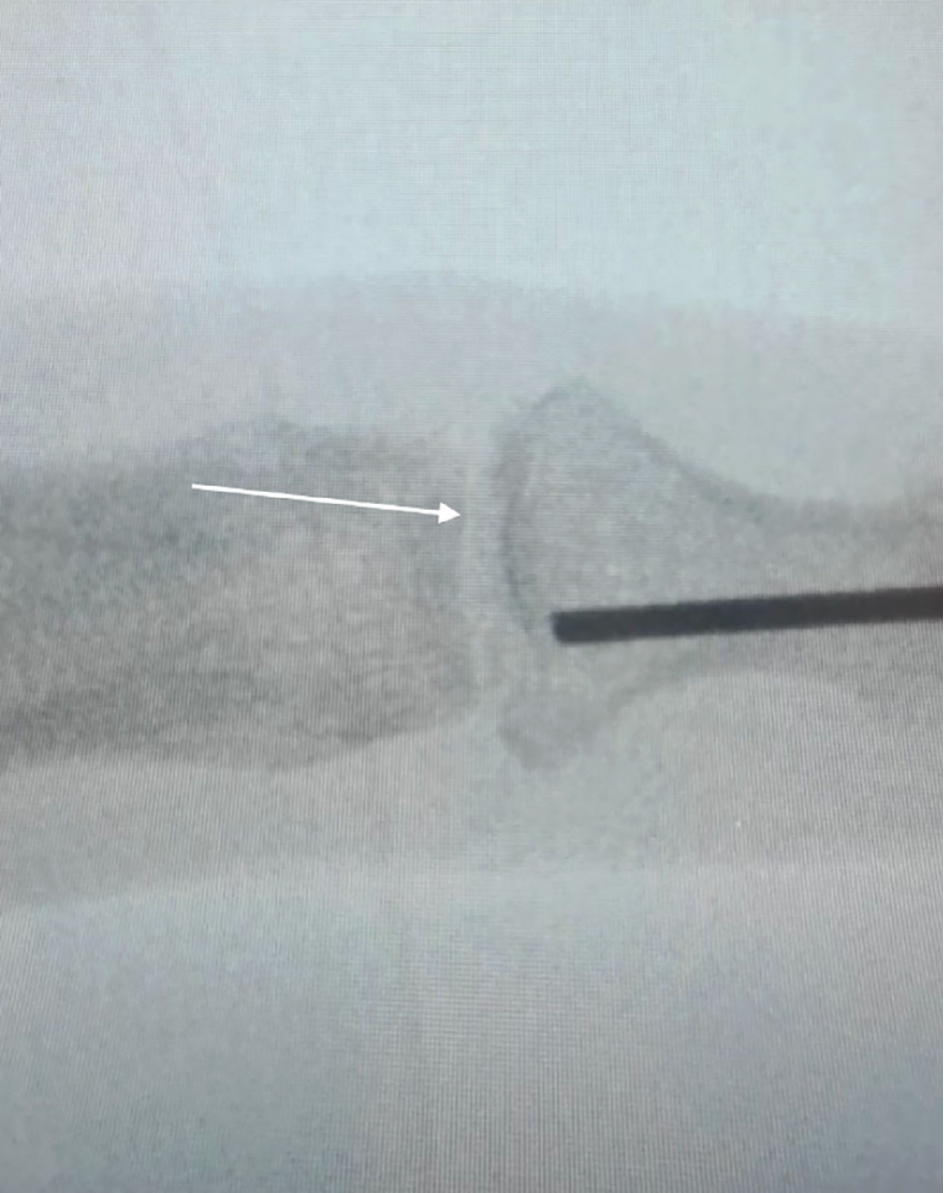
Intraoperative radiograph showing successful indirect reduction of DIP joint (white arrow) using the K‐wire as a bone tamp.

1. The patient was put under general anaesthesia and positioned supine with the affected hand on an arm table. The surgical site was cleaned and draped using sterile technique, and a tourniquet was applied to the left arm.
2. Indirect reduction of the DIP joint was achieved by retrograde drilling of a 1.1 mm K‐wire through the tuft towards the joint surface (Fig. 1). After trepanation of the cortex, the wire was reversed, using the blunt end and applied like a bone tamp to reduce the articular surface (Figs. 2 and 3).
3. The intra‐articular fracture was subsequently stabilized by percutaneously introducing two orthogonal 1.2 mm screws, one in the posteroanterior direction and the other in the radio‐ulnar direction.
4. Appropriate reduction and fixation of the DIP joint were confirmed through intraoperative radiography with the K‐wire *in situ*.
5. The incision sites were closed using a 4–0 vicryl rapide suture and dressed with jelonet gauze. A mallet splint was applied until bony union at 4 weeks.

## Discussion

The use of indirect reduction and percutaneous orthogonal screws provided the benefits of stable fracture fixation, and minimized soft tissue and joint disruption.^7^, ^8^ Moreover, the technique of using the K‐wire as a bone tamp, like the treatment of tibial plateau fractures, minimizes damage to surrounding soft tissues and allows for buried percutaneous stabilization, which reduces the risk of infection.^6^ The combined use of the K‐wire for indirect reduction and orthogonal screws for fixation offers the joint benefits of reduced soft tissue complications, prevention of joint stiffness, and early active joint mobilization.^6^, ^7^, ^8^ This approach reduces surgical complications and facilitates early recovery with full active range of movement.

## Author contributions

**Laurent Willemot**: Writing ‐ review & editing; methodology; supervision; writing ‐ original draft. **Timothy Marshall**: Writing ‐ review & editing; methodlogy; conceptualization; supervision. **Him Shun Yuen:** Writing ‐ review & editing; writing ‐ original draft.
